# Systematic Review and Meta-Analysis of Prevalence of Depression Among Caregivers of Cancer Patients

**DOI:** 10.3389/fpsyt.2022.817936

**Published:** 2022-05-12

**Authors:** Yuan-Chien Pan, Yaw-Sheng Lin

**Affiliations:** Department of Psychology, National Taiwan University, Taipei, Taiwan

**Keywords:** meta-analysis for depression among caregivers depression, caregivers, cancer patients, prevalence, meta-analysis, depression

## Abstract

**Background:**

Caregivers of cancer patients commonly experience depressive symptoms due to the heavy burden of caregiving responsibility.

**Objective:**

This meta-analysis examined the prevalence of depression among caregivers of cancer patients.

**Methods:**

We included 85 studies covering 23,317 participants published between 2001 and 2021 (25 countries) that reported the prevalence of depression among caregivers of cancer patients. We examined the pooled prevalence of depression and hypothesized moderators, including year, age, sex, geographic regions, percentage of spousal caregivers, depression measures, and cancer stage.

**Results:**

All 85 effect sizes included 6,077 caregivers of patients with depression. The weighted average prevalence of depression was 25.14% (95% CI, 21.42–29.27%) among caregivers. The prevalence rates were moderated by geographic region, patients' cancer stage, and measures for depression. The prevalence rates also varied among the different measures assessing depression. The prevalence rate decreased with the mean age of the caregivers and the percentage of spousal caregivers.

**Conclusions:**

This study revealed a high prevalence of depression among caregivers of cancer patients. The prevalence rates also varied with the study design, demographics of caregivers, and patients' medical information. These findings highlight that psychological support and intervention may be crucial for patients and their caregivers in clinical practice.

## Introduction

Day-to-day caregiving responsibilities are a heavy burden for caregivers of patients suffering from life-threatening illnesses, such as cancer. Additionally, caregivers must contend with their fear and distress regarding their loved ones' health and prognosis. In the past two decades, a growing body of research has documented elevated levels of psychological distress among caregivers. Previous studies suggested that routinely caregiving to a close family member with cancer may negatively affect physical and mental health (1, 2). A wide range of psychosocial, physical, and economic factors contribute to family caregivers' burden throughout the patient's illness, from diagnosis to possible death (3). Several studies have found that a high proportion of family caregivers suffer from depressive symptoms (4, 5). Caregivers' depressive symptoms are also associated with worse quality of life, physical well-being, and less leisure time (4). Depressive symptoms can also compromise caregivers' ability to effectively maintain their role (e.g., providing day-to-day care and satisfying the psychological needs of patients). Thus, investigating the prevalence of depression among caregivers is crucial for enhancing their well-being and improving the quality of caregiving.

In previous studies, the depression prevalence rates among caregivers of cancer patients had significant variance, ranging from 2.6 to 82.2% (6). A community study showed that caregivers of cancer patients scored significantly higher for depression than the UK adult general population. Almost 25% of all caregivers are evaluated as having moderate, severe, or even highly severe depression (7). A meta-analysis included 30 studies with 21,149 participants to estimate the depression prevalence in caregivers of cancer patients; the pooled depression prevalence rate was 42.3% (6). The meta-analysis also identified several factors associated with depressive symptoms, primarily related to caregiver characteristics, such as caregiving burden, spousal caregiving, sex of the caregiver, and caregiver's age. In another meta-analysis that included 58 studies with 9,262 parents of children with cancer, the pooled prevalence of depression was 28.0%, with high heterogeneity. The depression prevalence was also higher than in parents of children without cancer (8).

The heterogeneity of the depression prevalence rates among caregivers may also depend on other moderators, such as geographic regions, depression measurements, and patients' cancer stage. In Denmark, a nationwide population-based cohort study (*N* = 2,865) found that 17.2% of the family caregivers reported moderate-to-severe pre-loss depressive symptoms (9). In another nationally representative survey among caregivers of cancer patients in Korea, the prevalence rate of moderate-to-severe depression was 41.8% (10). Additionally, assessment tools for depression may account for divergent prevalence rates. In a previous meta-analysis, the pooled prevalence rates estimated with different measures ranged from 3.35% (with diagnostic criteria of depression in the DSM-IV) to 50.82% (with the Center for Epidemiological Studies Depression [CESD]). A meta-analysis examined the prevalence of anxiety, depression, and post-traumatic stress disorder (PTSD) among caregivers of children with cancer. The high heterogeneity was not explained by the caregiver's sex or child's cancer phase but was likely due to significant methodological differences in measurement tools and defined thresholds among the three psychiatric disorders (8). Furthermore, patients' medical information, such as the stage of cancer, may also affect the prevalence of depression in their caregivers. Several studies have found that the cancer stage can significantly explain variations in the severity of depressive symptoms (11–13). Contrastingly, a recent Asian study found no association between patients' cancer stage and caregivers' depression (14). Considering that the characteristics of caregivers and study design may highly impact the prevalence of depression in caregivers of patients with cancer, a more comprehensive meta-analysis is urgently needed.

Even among caregivers of patients with other illnesses, depression is a common experience and the caregivers' characteristics and methodology moderate the depression prevalence rates. In a meta-analysis examining the prevalence of mental health disorders among caregivers of patients with Alzheimer's disease, the pooled prevalence of depression among caregivers was 34.0%. The meta-analysis also revealed that the odds of depression were 2.51 times higher in spousal caregivers (15). A meta-analysis investigated the association between subjective caregiver burden and depressive symptoms among the caregivers of older relatives. They found that care for patients with illness and the sampling method accounted for 45.0% of the heterogeneity. Sex, mean age of caregivers, and percentage of spouses of caregivers did not contribute to the meta-regression model (16). A recent meta-analysis examining the prevalence of depression among informal caregivers of people with dementia revealed that depression prevalence differed according to the instrument used to assess depression, with interviews based on diagnostic criteria yielding the lowest pooled prevalence estimate (17). Due to the impact of these factors on the prevalence of depression in caregivers, it is necessary to conduct a meta-analysis to re-examine the moderators of depression prevalence in caregivers.

Therefore, there is a need to examine the depression prevalence in caregivers of cancer patients and their corresponding moderators. Our primary aim was to determine the pooled prevalence rates of depression among caregivers worldwide. Moderators of interest included 1) characteristics of study: year of publication, geographic regions (i.e., Eastern vs. Western sample), and measures of depressive symptoms; 2) demographics of caregivers (i.e., mean age of caregivers, proportion of male caregivers, and percentage of spousal caregivers); and 3) patients' medical information (i.e., cancer stage).

## Methods

### Search Strategy

**Figure 1:**
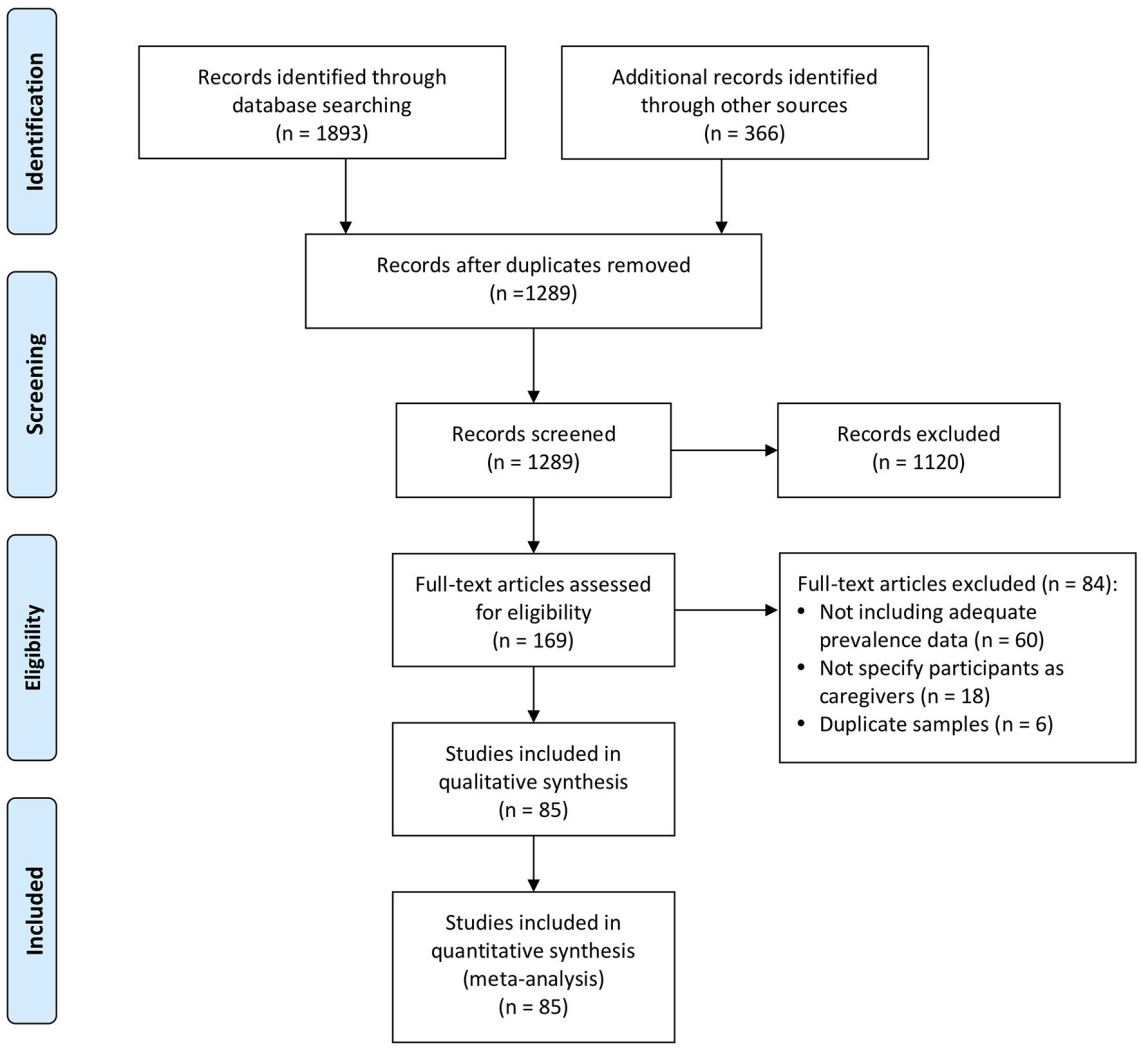
PRISMA flow diagram of the review process.

PubMed and PsycINFO were searched using the following search terms with combinations (Caregiver OR Carer OR Relatives [tw] OR Famil^*^ OR Spouse [tw] OR Parent [tw]) AND (Cancer OR Oncology OR Tumor) AND (Depression) AND (Epidemiology OR Prevalence). Reference lists from related articles were included in other relevant studies. Articles published in English that explored depression in caregivers of cancer patients were initially included. Studies involving caregivers who cared for illnesses other than cancer (e.g., rare diseases or dementia) were excluded. Studies were included if they reported the number or percentage of participants with depression assessed by diagnostic interviews based on various criteria. These included the Diagnostic and Statistical Manual of Mental Disorders (DSM), the International Classification of Disease (ICD), or validated self-report measures with specified clinical cutoffs. Two reviewers came to a consensus decision regarding each study wherein there was ambiguity regarding inclusion. Figure 1 shows the flow diagram of the literature search. The original articles included in the identification phase were published between 1972 and 2021. When more than one study reported the same sample, we chose the effect size associated with the most recent and complete data from a given study. The search was updated in August 2021.

### Study Selection

All studies included in this study met the criteria described by the participants, intervention, comparison, outcome, and study design (PICOS). Participants (P): informal caregivers of cancer patients; Intervention (I): not applicable; Comparison (C): not applicable; Outcomes (O): reported data for depression as defined by self-reported scales with well-validated cutoff values, self-reported medical history, or diagnostic interviews; and Study design (S): all included articles were epidemiological surveys or empirical studies with prevalence data for depression. Data on the depression prevalence in cancer patient caregivers, sample demographic data, country variables, percentage of spousal caregivers, assessment tools, the cutoff point of assessment tools, and patients' medical information were coded. The meta-analysis adhered to the Preferred Reporting Items for Systematic Reviews and Meta-Analyses guidelines (PRISMA) (18).

### Meta-Analysis

We used the metaphor and metaviz package in R to meta-analyze the data (19, 20). The prevalence rates were transformed using logit transformation to normalize the data distribution with inverse variance weighting (21). A random-effects model was used to estimate the average weighted prevalence of depression. We also calculated the depression prevalence rates for each geographic region separately. Cochran's *Q* statistic was used to assess whether the prevalence rates were homogeneous across samples. The estimates' heterogeneity across studies was examined using *I*^2^ index (21). *I*^2^ values above 50 and 75% indicate heterogeneity and high heterogeneity, respectively (22). Mixed-effects meta-regression was used to test whether the depression prevalence changed across regions or other design predictors. Meta-regression analyses were then performed to identify moderators that explained the heterogeneity of prevalence.

## Results

The search netted 85 studies from 25 different countries, conducted between 2001 and 2021, covering 6,077 cases of depression, with 23,317 participants (5, 9, 10, 14, 23–103). Table 1 lists all studies included in the analyses. There are 85 effect sizes obtained from these 85 studies, which were all on informal caregivers.

**Table 1:** Studies included in meta-analysis.

**First Author**	**Year of Publication**	**Prevalence**	**Total N**	**Location**	**Region (East/West)**	**Measures for depression**
Applebaum	2020	14.00%	14	US	W	HADS
Areia	2018	68.80%	112	Portugal	W	Other
Baudry	2019	15.00%	364	France	W	HADS
Bonafede	2020	32.98%	94	Italy	W	BDI
Bradley	2004	25.90%	174	US	W	SCID
Burns	2021	48.65%	74	Russia	W	DASS-21
Carey	2017	21.00%	989	Australia	W	DASS-21
Corà	2012	45.00%	20	Italy	W	BDI
Costa-Requena	2012	77.40%	159	Spain	W	HADS
Doubova	2016	6.80%	826	Mexico	W	HADS
Duckworth	2020	39.00%	58	US	W	CESD
Fancourt	2019	11.50%	62	UK	W	HADS
Fasse	2015	25.00%	60	France	W	BDI
Fenix	2006	28.00%	175	US	W	SCID
Fletcher	2008	12.20%	60	US	W	CESD
Goren	2014	16.00%	1,713	European	W	Self-reported diagnosis
Gough	2009	12.26%	106	Australia	W	HADS
Grov	2005	23.96%	96	Norway	W	HADS
Gupta	2021	28.60%	904	US	W	PHQ
Haj Mohammad	2015	2.00%	47	Netherland	W	HADS
Haley	2001	52.50%	40	US	W	CESD
Han	2013	47.20%	301	China	E	CESD
Haun	2014	27.00%	189	Germany	W	PHQ
He	2020	25.50%	98	China	E	Other
Heckel	2015	30.00%	150	Australia	W	CESD
Hyde	2018	9.00%	427	Australia	W	HADS
Jamani	2018	14.80%	849	Canada	W	Self-reported diagnosis
Janda	2017	14.00%	84	Australia	W	HADS
Janda	2008	10.30%	70	Australia	W	HADS
Jassem	2015	17.80%	107	European	W	Self-reported diagnosis
Kang	2021	24.38%	159	South Korea	E	PHQ
Katende	2017	26.00%	119	Uganda	W	HADS
Kehoe	2019	18.90%	414	US	W	PHQ
Kim	2014	20.00%	60	US	W	CESD
Kim	2008	24.60%	314	US	W	CESD
Kim	2005	30.00%	120	US	W	CESD
Kleijn	2020	29.70%	64	Netherland	W	HADS
Krähenbühl	2007	21.60%	109	Germany	W	Other
Lambert	2013	5.20%	436	Australia	W	HADS
Langenberg	2019	7.00%	61	Netherland	W	HADS
Lee	2017	14.69%	143	Taiwan	E	SCID
Lee	2013	17.00%	106	Taiwan	E	HADS
Leroy	2016	45.00%	60	France	W	HADS
Lin	2020	31.53%	555	China	E	HADS
Ling	2013	60.00%	217	Taiwan	E	CESD
Lutfi	2019	72.00%	250	Iraq	E	BDI
Malpert	2015	3.10%	127	US	W	Other
Mazanec	2017	25.00%	12	US	W	Other
Mazzotti	2012	52.98%	151	Italy	W	HADS
Mekonnen	2020	23.30%	275	Ethiopia	W	BDI
Moser	2013	32.84%	137	Switzerland	W	HADS
Nielsen	2017	17.20%	2,865	Denmark	W	BDI
Nik Jaafar	2014	33.90%	130	Malaysia	E	DASS-21
Oberoi	2016	11.00%	196	Australia	W	HADS
Oechsle	2019	39.00%	232	Germany	W	PHQ
Oechsle	2013	21.21%	33	Germany	W	PHQ
Papastavrou	2009	66.40%	130	Greek	W	CESD
Park	2018	88.00%	100	South Korea	E	HADS
Park	2013	41.81%	897	South Korea	E	HADS
Pawl	2013	28.00%	126	US	W	CESD
Price	2009	5.10%	373	Australia	W	HADS
Rhee	2008	66.80%	310	South Korea	E	BDI
Rumpold	2017	17.90%	80	Austria	W	HADS
Rumpold	2015	21.30%	345	Austria	W	HADS
Sacher	2018	31.00%	45	Germany	W	CESD
Sahadevan	2019	18.00%	384	India	E	Other
Schellekens	2016	7.10%	98	Netherland	W	SCID
Shaffer	2017	22.80%	491	US	W	CESD
Simpson	2015	9.80%	51	Australia	W	HADS
Stenberg	2014	24.30%	278	Norway	W	CESD
Sterba	2017	37.00%	72	US	W	CESD
Stieb	2018	20.00%	50	Germany	W	HADS
Tang	2021	52.10%	309	Taiwan	E	CESD
Tang	2013	54.90%	193	Taiwan	E	CESD
Toledano-Toledano	2021	45.70%	330	Mexico	W	BDI
Trevino	2018	4.10%	540	US	W	SCID
Vanderwerker	2005	4.50%	200	US	W	SCID
Virtue	2014	18.00%	215	US	W	SCID
Waldman	2021	6.20%	193	US	W	HADS
Wang	2016	28.20%	117	China	E	BDI
Wen	2021	79.60%	661	Taiwan	E	CESD
Williams	2014	21.30%	150	US	W	CESD
Yang	2021	8.90%	191	China	E	HADS
Yang	2012	63.50%	312	China	E	CESD
Yu	2017	21.00%	309	China	E	HADS

*E, Eastern country; W, Western country; CESD, Center of Epidemiological Studies Depression Scale; HADS, Hospital Anxiety and Depression Scale; BDI, Beck Depression Inventory; DASS-21, Depression Anxiety Stress Scale; PHQ, Patient Health Questionnaire; SCID, Structured Clinical Interview for DSM*.

**Figure 2:**
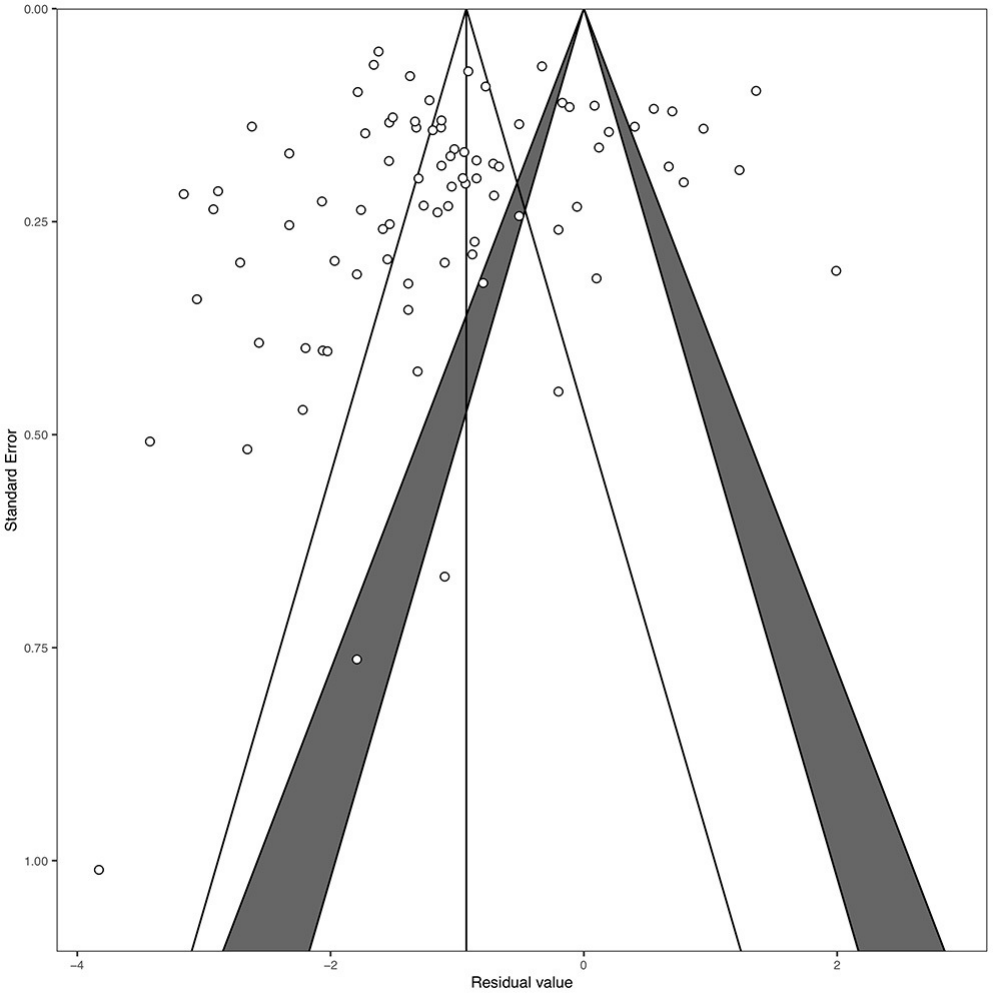
Funnel plot for prevalence of depression among caregivers of cancer patient.

**Figure 3:**
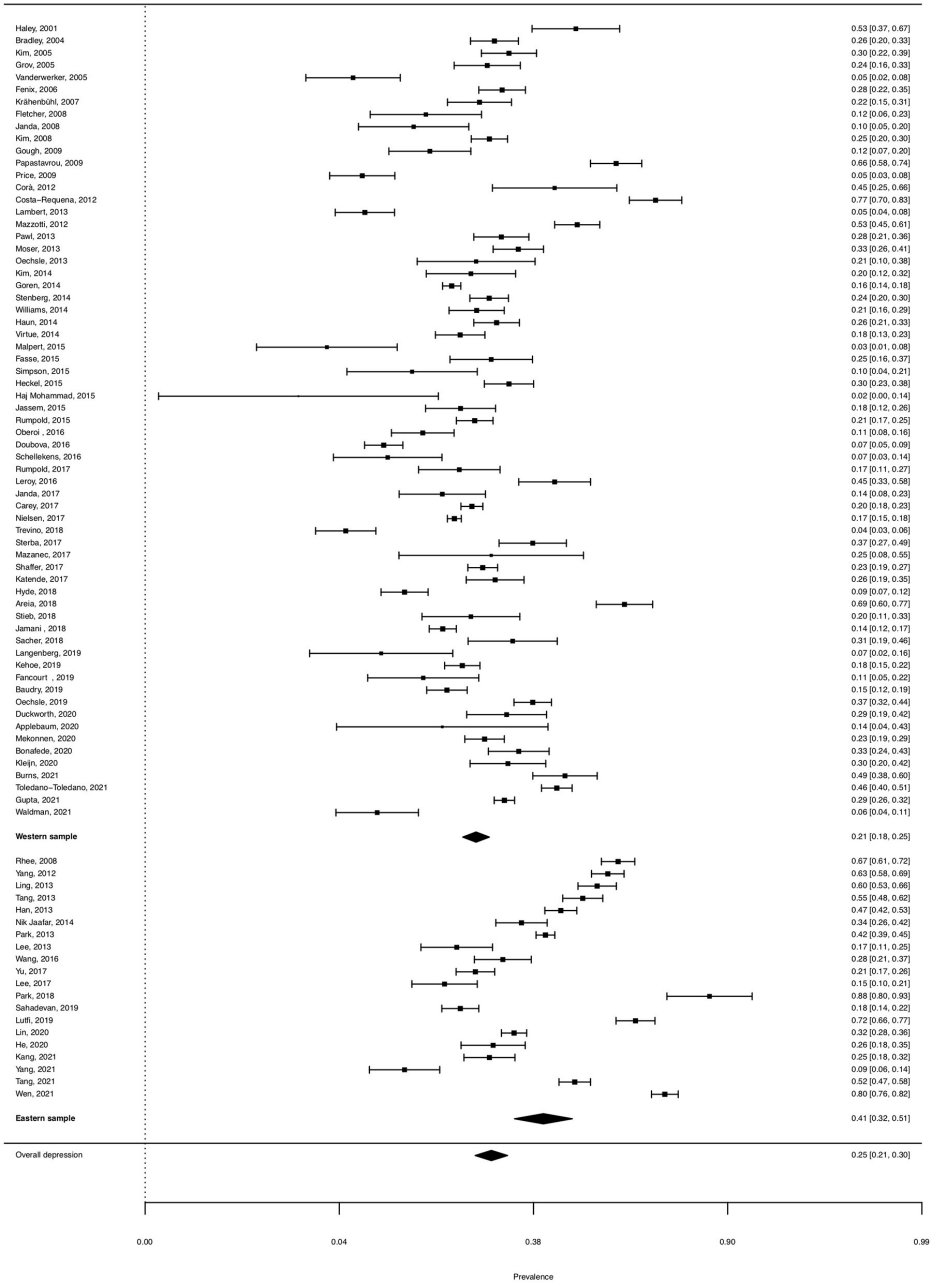
Forest plot for prevalence of depression among caregivers of cancer patient.

Funnel plot and Egger's test indicated no publication bias (*t* = −0.66, *df* = 83, *p* = 0.508, Figure 2). The pooled prevalence of clinical depression was 25.14% (95% CI, 21.42–29.27%). There was significant heterogeneity between the studies (*Q*_*E*_ = 3249.96, *df* = 84, *p* < 0.0001; *H*^2^ = 38.69, *I*^2^ = 97.42%). Forest plots of the estimated depression prevalence by study are shown in Figure 3.

### Meta-Regression Analyses

Table 2 shows depression prevalence and the effect of hypothesized moderators. The depression prevalence significantly decreases with the mean age of the caregivers (β = −0.037, *p* = 0.007). The prevalence of depression significantly decreases with the percentage of spousal caregivers (β = −1.753, *p* = 0.007). The pooled prevalence is 41.09% for the Eastern sample and 21.19% for the Western sample. The prevalence rate of the studies conducted in the Eastern countries is significantly higher (β = −0.969, *p* < 0.001) than the prevalence rate in Western countries. The year of publication (β = −0.002, *p* = 0.925) and proportion of male caregivers (β = 0.152, *p* = 0.864) did not significantly explain the variance in the depression prevalence.

**Table 2:** Clinical depression prevalence by geographic region and measures.

**Subgroups**	**Categories**	***k***	**Prevalence (%)**	**95% CI (%)**	***I^**2**^***	***Q***	***p*-value^**a**^**
Clinical depression		85	25.14	21.42 – 29.27	97.42%	3249.96	<0.001
Geographic region	Eastern sample	20	41.09	31.65 – 51.25	97.92%	913.77	<0.001
	Western sample	65	21.19	18.21 – 24.52	95.13%	1314.39	<0.001
Patient stage of cancer	Non-advanced cancer	55	22.14	18.31 – 26.52	96.85%	1713.41	<0.001
	Advanced cancer	12	18.83	9.66 – 33.48	98.52%	742.04	<0.001
	Terminal or palliative stage	18	41.64	30.05 – 54.24	97.73%	748.94	<0.001
Measures	SCID	7	12.12	6.61 – 21.17	94.03%	100.43	<0.001
	CESD	20	38.46	29.31 – 48.50	97.10%	655.51	<0.001
	HADS	31	18.79	13.65 – 25.28	96.74%	920.30	<0.001
	BDI	9	38.32	22.58 – 56.96	98.67%	602.31	<0.001
	DASS-21	3	32.71	18.24 – 51.44	94.56%	36.79	<0.001
	PHQ	6	26.19	20.92 – 32.25	84.00%	31.24	<0.001
	Other self-reported tools	6	22.75	10.23 – 43.24	95.73%	117.22	<0.001
	Self-reported diagnosis	3	15.57	14.24 – 17.00	0.00%	1.55	0.450

*SCID, Structured Clinical Interview for DSM; CESD, Center of Epidemiological Studies Depression Scale; HADS, Hospital Anxiety and Depression Scale; BDI, Beck Depression Inventory; DASS-21, Depression Anxiety Stress Scale- 21-itens; PHQ, Patient Health Questionnaire. The “a” indicates the p-value stands the significant heterogeneity*.

For the different measures, the pooled prevalence was 38.46% for the Center of Epidemiological Studies Depression Scale (CESD), 18.79% for the Hospital Anxiety and Depression Scale (HADS), 38.32% for the Beck Depression Inventory (BDI), and 32.71% for the Depression Anxiety Stress Scale (DASS-21). Further, 26.19% for the Patient Health Questionnaire (PHQ), 12.12% for the Structured Clinical Interview for DSM (SCID), 22.75% for other self-reported tools (i.e., other validated instruments for assessing depression), and 15.57% for self-reported diagnosis (i.e., the respondents' self-reports about whether they had been diagnosed with depression). Additionally, the pooled prevalence rate was significantly higher in studies using CESD (β = 1.514, *p* < 0.001) and BDI (β = 1.510, *p* = 0.002) than in SCID. In terms of caring for cancer patients, the depression prevalence was 22.14% for caring for patients with non-advanced cancer, 18.83% for caring for those with advanced cancer, and 41.64% for caring for those with terminal or palliative stage cancer. The depression prevalence was significantly lower in studies involving caregivers caring for non-advanced cancer (β = −0.930, *p* < 0.001) and advanced cancer (β = −1.107, *p* = 0.004) than in studies involving caregivers caring for terminal or palliative cancer stages.

## Discussion

To the best of our knowledge, this is the first meta-analysis to examine geographical regions, caregiver characteristics, and patients' cancer stage as moderators of the depression prevalence among cancer patient caregivers. We extended previous studies on the depression prevalence in caregivers (6) to evaluate 85 prevalence rates based on studies from 25 countries from 2001 to 2021. Our primary findings showed that the pooled depression prevalence rate was 25.14% among cancer patient caregivers. Only one meta-analysis investigated the depression prevalence in cancer patient caregivers and found that the pooled depression prevalence was 42.30% (6). This difference could be because the previous meta-analysis did not use “prevalence” or “epidemiology” as keywords in the literature search stage. Instead, they included studies evaluating the depression prevalence based on self-reported measures with loosened cutoff points. Additionally, the current meta-analysis included almost three times as many studies and may have provided a more accurate prevalence estimate. Considering moderators for depression prevalence, the pooled prevalence rate estimated from the Eastern sample was significantly higher than that estimated in the Western sample. Furthermore, the depression prevalence rate was higher in studies involving caregivers caring for patients with terminal or palliative cancer than in studies involving caregivers caring for patients with non-advanced and advanced cancer. The current meta-analysis also revealed that the depression prevalence rates differed according to the measures used to assess it. In addition, the prevalence rates of depression significantly decreased with the mean age of caregivers and percentage of spousal caregivers.

The pooled prevalence of depression among caregivers found in the current meta-analysis was lower than that found in caregivers caring for other illnesses. For example, prevalence studies have shown that depressive symptoms in caregivers of older relatives are 40.2% for those caring for stroke survivors (104) and 34.0% for caregivers of people living with AD (15). Moreover, a meta-analysis examined the prevalence of depression among caregivers of individuals with dementia. They found that the pooled depression prevalence was 31.24% (17). Compared to the general adult population, several studies have directly compared the prevalence of depressive symptoms between caregivers and community-dwelling adults. For example, a UK study found that caregivers scored considerably higher for depression compared to the adult general population sample, with almost 25% of all caregivers being moderately, severely, or even highly severely depressed (7). Additionally, a meta-analysis evaluated the depression prevalence in communities in 30 countries between 1994 and 2014. They found that the depression prevalence among adults in the community was 12.9% (105). Further, it was lower than the pooled prevalence in the present study in the general population. This implies that caregivers experience more physical problems and psychological burdens. Our findings demonstrate that cancer patient caregivers may need long-lasting interventions compared to the general adult population.

The significantly high heterogeneity in depression prevalence can be explained by several moderators. First, geographic regions may account for the heterogeneity in the prevalence rates of depression. The pooled prevalence rate estimated from the Eastern sample was significantly higher than that estimated in the Western sample. Second, the heterogeneity was partially explained by the instruments used to measure depression, with studies using diagnostic interviews of depression yielding lower pooled prevalence estimates. Most of the studies included in the current meta-analysis used self-report tools to estimate the prevalence of depression. Mental health care specialists should keep in mind that the prevalence rates of depression among caregivers may depend highly on the assessment tools they used. This is also consistent with the findings from previous meta-analyses focused on the depression prevalence among caregivers (6, 17). The discrepancy in depression prevalence rates evaluated from studies using self-report instruments and from studies using diagnostic interviews should be considered by mental health professionals working with caregivers. Additionally, heterogeneity was explained by the patients' cancer stage. The prevalence rate of depression was higher among studies involving caregivers caring for patients with terminal or palliative cancer than those involving caregivers caring for patients with non-advanced and advanced cancer.

The current meta-analysis showed that caregivers in Eastern countries had a higher depression prevalence than caregivers in Western countries. Such cultural discrepancies were not examined in any previous meta-analysis of cancer patient caregivers. Further, a previous study found that cancer patient caregivers in Asia reported a significantly lower quality of life than caregivers in Western countries (106). They suggested that this may have been influenced by filial piety and obligatory care. A study investigating the caregiving burden among Asian caregivers of dementia patients identified a unique dimension described as “worry about caregiving performance” (107). When caring for patients with cancer, it is essential to recognize that caregivers' cultural roots or social norms may determine their beliefs about caring for them and the burden they received. Considering the relationship between caregivers and patients, we found that the prevalence of depression decreased as the percentage of spousal caregivers increased. A previous study also found that adult–child caregivers reported higher scores on the depression scale than spousal caregivers (108). Other studies also found low levels of depression among spousal caregivers of cancer patients (109, 110). Additionally, spousal caregivers reported feelings of loneliness and loss (111). Non-spousal caregivers reported a higher depression and perceived burden rate than other caregivers. Nevertheless, this finding could have been attributed to sex and cultural differences (112).

In line with previous meta-analyses examining depression prevalence among caregivers (6, 16, 17), the current meta-analysis found that the depression prevalence rates differed according to the measures used to assess depression. In this study, studies using SCID reported a significantly lower prevalence of depression than those using the CESD and BDI. In addition, consistent with our findings, a previous meta-analysis of caregivers of cancer patients also found that the prevalence of depression evaluated with diagnostic interviews yielded the lowest pooled prevalence estimate (6). Another meta-analysis revealed that the depression prevalence estimates differed according to the measures used. They found that two studies that used diagnostic criteria reported the lowest prevalence rate, and those using the BDI reported the highest prevalence estimate (17). Divergent prevalence rates were discovered in caregivers and the general population. A meta-analysis examined the depression prevalence in community samples and found that most studies using self-report instruments were significantly higher than those using interview-based assessment tools (105). The differences in the assessment tools could explain the heterogeneous prevalence rates for depression and the cutoffs used.

Our results revealed that depression among cancer patient caregivers is highest in studies with caregivers of patients with terminal cancer or the palliative stage, compared to those caring for non-advanced or advanced cancer. Several empirical studies have found that the patients' cancer stage is associated with depressive symptoms and quality of life among caregivers (62, 63, 106). The impending death of a close family is not unexpected (113). As caregivers often have to deal with role changes and new caregiving tasks, they can experience caregiver burden, which has been shown to influence depression (114). Recently, a systematic review investigated the associations between pre-loss grief and post-loss adjustment among caregivers of patients with terminal cancer. They found that high levels of pre-loss grief and low levels of perceived preparedness for death were associated with prolonged grief and more depressive symptoms (115). In the terminal or palliative stage, the depressive symptoms of caregivers may overlap with anticipatory grief and move from curing the patient's illness toward facing the patient's death soon.

Several methodological limitations should be noted. First, some factors were not reported in sufficient detail to be included as moderators, such as duration of caregiving and time since diagnosis. Future studies should consider the impact of such factors that may affect the prevalence of depression. Second, our search strategy may have also neglected some studies that reported the proportion of depression but did not use the word “prevalence” or “epidemiology” in the abstract. These strategies may avoid enhancing heterogeneity but may also omit potential prevalence data. Third, although the current study tried to use a rigorous database searching strategy, which is essential for systematic review, it may still have ignored potential studies which only include other alternative terms. Furthermore, future meta-analyses focused on caregivers of cancer patients and other mental health conditions are also urgently needed.

In conclusion, the prevalence of depression among caregivers of patients with cancer varied with the age of the caregivers and the percentage of spousal caregivers. The prevalence rates were also moderated by geographic region, patients' cancer stage, and measures for depression. This meta-analysis revealed that almost one-fourth of the caregivers of cancer patients experience depression. This highlights that psychological support may be necessary for cancer patients and their caregivers. Several sociodemographic and care-related risk factors impact caregivers' mental burden and quality of life. Therefore, it is crucial to move forward from a patient-centered approach to a family-centered approach to reduce the burden on family caregivers when facing the imminent death of a family member and prevent the deterioration of depressive symptoms.

## Data Availability Statement

The original contributions presented in the study are included in the article/supplementary material, further inquiries can be directed to the corresponding author.

## Author Contributions

Y-SL and Y-CP contributed to conception, design of the study, and organized the database. Y-CP performed the statistical analysis and wrote the first draft of the manuscript. Both authors contributed to manuscript revision, read, and approved the submitted version.

## Funding

This study was supported by grants from the Ministry of Science and Technology, Taiwan (MOST 107-2410-H-002-119-MY3).

## Conflict of Interest

The authors declare that the research was conducted in the absence of any commercial or financial relationships that could be construed as a potential conflict of interest.

## Publisher's Note

All claims expressed in this article are solely those of the authors and do not necessarily represent those of their affiliated organizations, or those of the publisher, the editors and the reviewers. Any product that may be evaluated in this article, or claim that may be made by its manufacturer, is not guaranteed or endorsed by the publisher.
